# Enhanced quantitative method for the diagnosis of grade 1 cardiac amyloidosis in ^99^mTc-DPD scintigraphy

**DOI:** 10.1038/s41598-022-05689-8

**Published:** 2022-02-02

**Authors:** María del Carmen Mallón Araujo, Estephany Abou Jokh Casas, Charigan Abou Jokh Casas, Pablo Aguiar Fernández, María Amparo Martínez Monzonís, Bernardo Sopeña Pérez-Argüelles, Virginia Pubul Núñez

**Affiliations:** 1 University of Santiago de Compostela, Santiago de Compostela, Spain; 2 Present Address: Department of Nuclear Medicine, Santiago de Compostela University Hospital, Santiago de Compostela, Spain; 3 Department of Cardiology, Santiago de Compostela University Hospital, Santiago de Compostela, Spain; 4 Molecular Imaging and Medical Physics Group, Santiago de Compostela University Hospital, Santiago de Compostela, Spain; 5 Department of Internal Medicine, Santiago de Compostela University Hospital, Santiago de Compostela, Spain

**Keywords:** Molecular biology, Cardiology, Medical research

## Abstract

The lack of a standardized cut-off value in the quantitative method and an inter-observer disagreement in the evaluation of the semiquantitative score in ^99^mTc-DPD scintigraphy leaves several patients with cardiac amyloidosis (CA) undiagnosed (grade 1 and H/CL: 1–1.49). This study aims to increase diagnostic productivity of ^99^mTc-DPD scintigraphy in CA. This is a retrospective study of 170 patients with suspicion of CA. A total of 81 (47.6%) were classified as transthyretin CA (TTR-CA) and 9 (5.3%) as light-chain CA (LC-CA) applying the visual score. An enhanced quantitative method and cut-off point were attempted to reclassify inconclusive patients and reduce inter-observer variability. Applying the proposed quantitative method, of the 19 patients with grade 1 uptake, 2 became grade 0 (none-CA), 2 were reclassified as grade 3 (TTR-CA), and 2 were regrouped as grade 2 (1 TTR-CA and 1 LC-CA). Adjusting the quantitative method’s cut-off value to 1.3, four patients previously inconclusive were reclassified as TTR-CA, the diagnosis was confirmed in 3 and rejected in 1. When a 1.3 threshold is compared to 1.5, the sensitivity increases to 94% without reducing its specificity. The quantitative method improves the visual interpretation, reclassifying doubtful cases. The optimization of the cut-off value from 1.5 to 1.3 reclassifies a higher percentage of patients as TTR-CA with a higher sensitivity without reducing its specificity.

## Introduction

Cardiac amyloidosis (CA), considered until recently a rare and underdiagnosed disease, has been a known entity for more than 300 years; with an approximate incidence of 3 to 5 patients per million inhabitants per year and an average age of onset of around 65 years^1,2^. The term amyloidosis involves a heterogeneous group of subtypes (hereditary or acquired, localized or systemic)^2^, characterized by extracellular deposits of insoluble abnormal fibrils, which receive the pathophysiological name of amyloid deposits^3^. It can affect multiple organs, being the heart the second most affected in frequency (50–60%)^1^.

From the thirty known amyloid proteins, only a few have cardiac involvement, being the transthyretin (TTR) and the light chain (LC), the most frequently related to the heart^2,3^. Cardiac amyloidosis due to LC deposits is generally associated with lymphoproliferative disorders^1^ and has been considered to date the most prevalent disease form in developed countries. However, due to non-invasive diagnostic tests and the progressive aging of the population, amyloidosis due to TTR deposits (TTR) has become the most frequent^2^.

Until recently there was no specific treatment for this disease, mainly due to the need for histological tests such as an endomyocardial biopsy for its diagnosis and immunohistochemical examinations of the amyloid type. Currently, this pathology is at the center of attention, not only because of the advances in molecular imaging for its early diagnosis but also because of the recent approval of new therapeutic strategies aimed at transthyretin stabilizers in TTR-CA and chemotherapy in LC-CA. Therefore, the correct identification of the amyloid involved is vital to avoid toxic and inappropriate treatments^4^.

In 2016, Gillmore et al. published the results that allowed the design of the first diagnostic algorithm for a noninvasive diagnosis of TTR-CA, which did not require the performance of confirmatory invasive tests such as myocardial biopsy and allowed obtaining an earlier and safer diagnosis^3^. Thus, nowadays, ^99^mTc-DPD scintigraphy is known to accurately diagnose TTR-CA without cardiac biopsy^5^. For cardiac involvement assessment, various semiquantitative and quantitative methods have been proposed to differentiate TTR-CA from LC-CA. The best known and most used semiquantitative method is that of Perugini consisting of visual evaluation between cardiac and bone uptake, establishing four grades (from 0 to 3) depending on the absence of cardiac uptake or if it is less than, equal to, or greater than the bone, respectively^2^.

The most used quantitative analysis is the one proposed by Bokhari, who defined a heart/contralateral hemithorax ratio (H/CL) comparing the total number of counts obtained from a ROI plotted on the heart (H) divided by those obtained in the contralateral chest (CL), in images acquired 1 h after the administration of ^99^mTc-PYP (^99^mTc-pyrophosphate), establishing a cut-off value for the diagnosis of TTR-CA greater than 1.5^2^.

Based on these methods, a diagnostic algorithm declares that a visual assessment of grade 2 or 3 and a H/CL ratio ≥ 1.5 is indicative of TTR-CA, reaching a 100% specificity when associated with an absent monoclonal peak^4,6,7^. However, the diagnosis of CA is not easy, considering that 10–30% of patients with LC-CA have a cardiac uptake of 1 or 2, and that a monoclonal spike unrelated to CA can coexist in 40–50% of patients with TTR-CA^5,6^.

Despite diagnostic improvements, there are limitations such as the lack of a standardized cut-off point in the quantitative method and an inter-observer disagreement in the semiquantitative score, leaving a small yet critical number of patients undiagnosed, as they remain inconclusive or equivocal (visual uptake of grade 1), and therefore represent a real challenge^2,8,9^.

This study aims to increase the diagnostic productivity of ^99^mTc-DPD scintigraphy in CA, focusing on reclassification patterns, mainly those included in the category of inconclusive or equivocal, through the development and validation of an additional cut-off point value in the 3 h post-administration of ^99^mTc-DPD, complementing the Perugini visual score to provide more diagnostic certainty when interpreting cardiac scintigraphy and improve existing quantification methods.

## Materials and methods

### Study population and design

This study is a retrospective work of 170 patients, from 2016 to 2020, all patients were referred by the Cardiology and Internal Medicine departments, with a high clinical suspicion of CA. A total of 81 (47.6%) were TTR-CA and 9 (5.3%) LC-CA, the final diagnosis (gold standard) was reached through the international ASNC/AHA/ASE/EANM/HFSA/ISA/SCMR/SNMMI expert consensus recommendations, through clinical data, analytical parameters, electrocardiography, echocardiography, and sometimes cardiac or extracardiac tissue biopsy^10^.

From the 170 patients included in this study, 14 were submitted to biopsy confirmation. Most biopsies were performed on the heart (n: 4), followed by abdominal fat biopsy (n: 5), bone marrow (n: 4) and other locations (n: 3), also some patients were biopsied in more than one location. The results included 6 patients with TTR-CA, 2 with LC-CA, 5 with negative results and 1 with insufficient material.

The sample studied was similar to other registries, mostly consisted of men (N = 113; 66.5%) with a mean age of 78.3 ± 9.1 years. The Nuclear Medicine department performed a whole-body ^99^mTc-DPD scintigraphy study to all patients, acquiring images in anterior and posterior projections (3 h post-injection) and a planar oblique projection of the left anterior hemithorax in an OPTIMA 640™ NM/CT gamma camera. Also, a SPECT centered on the thoracic region was also acquired in all patients, using a 128 × 128 matrix, a low-energy high-resolution collimator, 15% energy window, 360 range, and CT attenuation correction. All methods were carried out in accordance with guidelines and regulations.

Informed consent was obtained from all the patients included, and the study was approved by the Ethics Committee in Clinical Research of the Santiago de Compostela University Hospital.

### Diagnostic performance

#### Visual score and quantitative method

The images were analyzed double-blinded by two nuclear medical observers, using two interpretation methods: the visual or Perugini semiquantitative method, and the quantitative method (heart/contralateral hemithorax ratio [H/CL]). The last one was obtained by drawing a circular ROI over the heart, then copied and mirrored over the contralateral chest; afterwards, the H/CL was obtained by dividing the total counts of the heart by the total counts in the contralateral hemithorax.

The patients were classified according to the ASNC practice points, into three groups: suggestive of TTR-CA when they presented a degree uptake of 2 or 3 or an H/CL index ≥ 1.5; inconclusive or equivocal with a degree 1 uptake or an H/CL index between 1 and 1.49, and not suggestive of TTR-CA with a degree of 0 uptake or an H/CL index < 1 Dorbala S et al. ^11^

#### Optimized cut-off point in the quantitative method

An additional quantitative method was determined and analyzed to complement the Perugini visual score, to reclassify inconclusive or equivocal patients with greater precision, and reduce the existing inter-observer variability. The reclassification consisted of a reappraisal of the diagnostic cut-offs of a well-established and well-validated scoring system, the heart to contralateral hemithorax ratio (H/CL), using the arithmetic mean and standard deviations in the study population within each Perugini grade. Finally, an additional cut-off point was sought to reclassify grade 1 CA to increase diagnostic yield and sensitivity and specificity of all methods were calculated.

### Statistical analysis

The descriptive and statistical analyses were carried out using the IBM SPSS Statistics 24 software. The descriptive analysis of the population was performed using categorical variables expressed as percentages and continuous variables as mean values with standard deviations. Kolmogorov–Smirnov tests were conducted for know if the data was normally distributed. Significance statistical tests and post-hoc analyses were carried out for demonstrating statistically significant differences between individual distributions. The Kruskal–Wallis test (non-parametric test) was used for studying significant differences between quantitative values of the different Perugini visual score groups. Post-hoc analyses were used for demonstrating statistically significant differences between individual groups.

### Patient consent

Patients have given their informed consent and permission to reproduce and distribute the information in this study.

## Results

### Visual score or semiquantitative analysis

Using the visual score proposed by Perugini for the diagnosis of CA, the patients were distributed as follows: 77 grade 0 (45.3%), 19 grade 1 (11.2%), 7 grade 2 (4.1%), and 67 grade 3 (39.4%).

**Figure 1:**
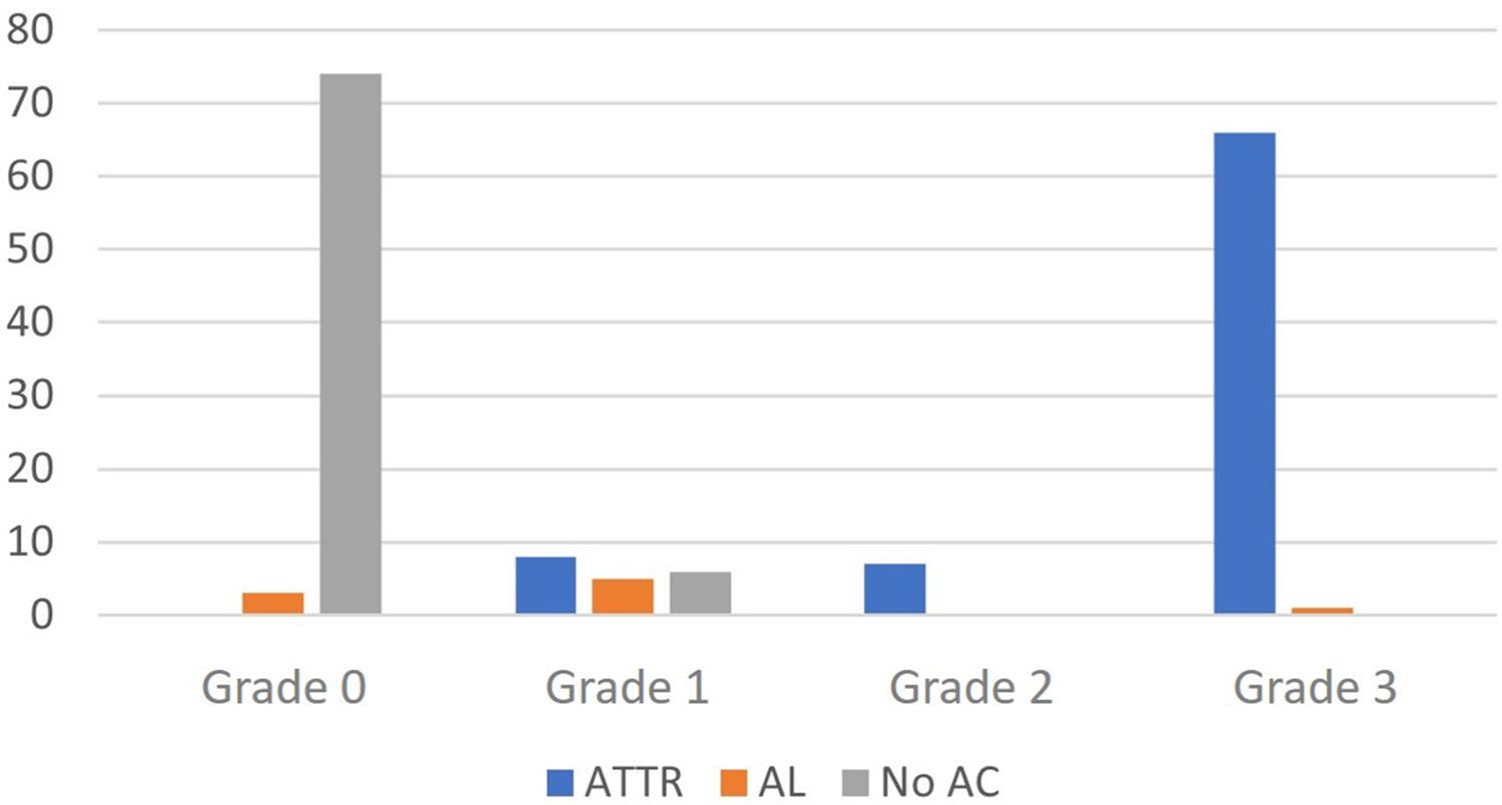
Relationship between the visual score and the definitive diagnosis of each patient.

**Figure 2:**
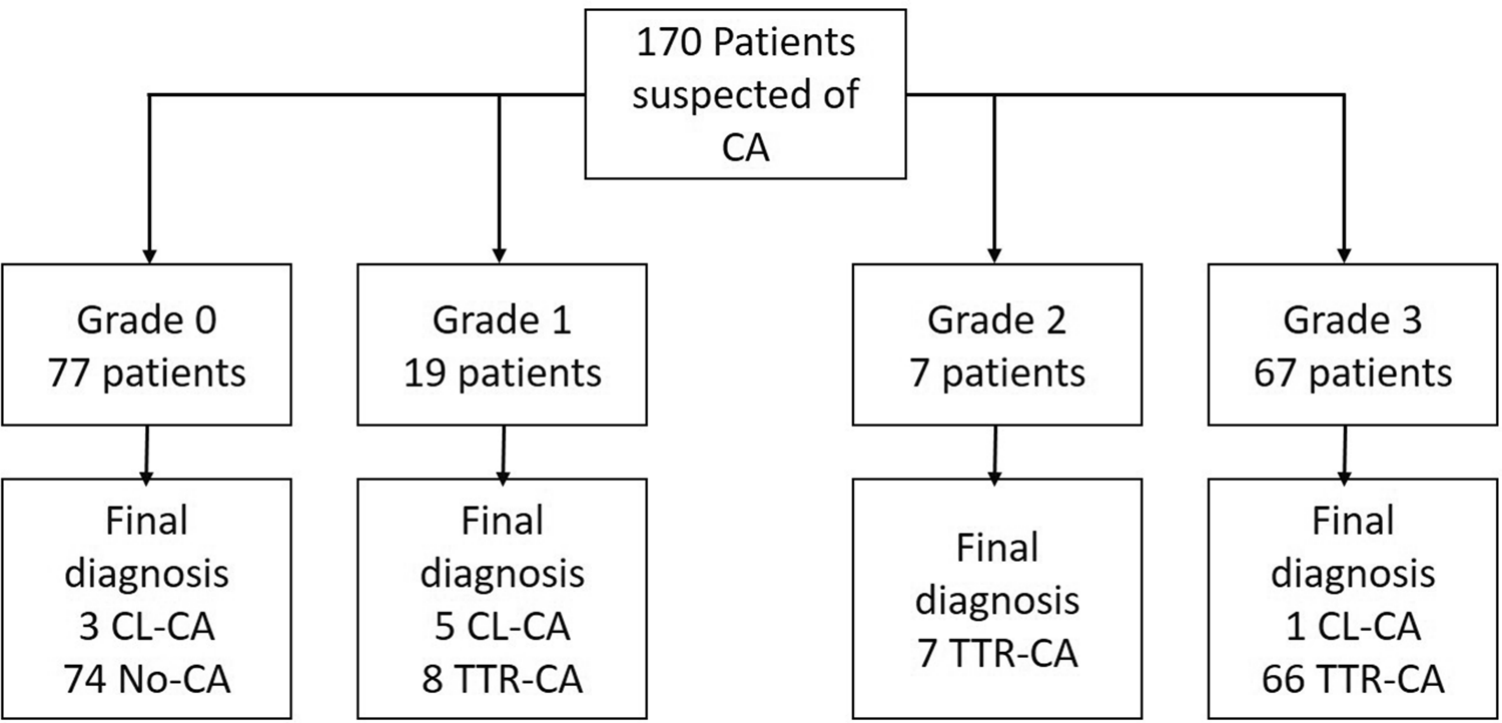
Patient’s flow chart in this study group.

When comparing this score with the final diagnosis, of the 81 (47.6%) patients with TTR-CA, 66 (81.5%) were classified as grade 3, 7 (8.6%) as grade 2, and 8 (9.9%) as grade 1. Of the 9 (5.3%) patients diagnosed with LC-CA, 5 (55.6%) were grade 1, 3 (33.3%) grade 0 and only 1 (11.1%) grade 3 (Fig. 1). In the cases where CA was ruled out, 6 (7.5%) were grade 1 and 74 (92.5%) had a grade 0 uptake (Fig. 2).

The visual score provides a sensitivity of 90% and a specificity of 99% (p < 0.05), considering the final clinical diagnosis (by international criteria) as the gold standard. Despite this, a considerable percentage of patients (n: 19; 11.2%) remained inconclusive, from a scintigraphic point of view.

### Additional quantitative method and optimized cut-off point

**Figure 3:**
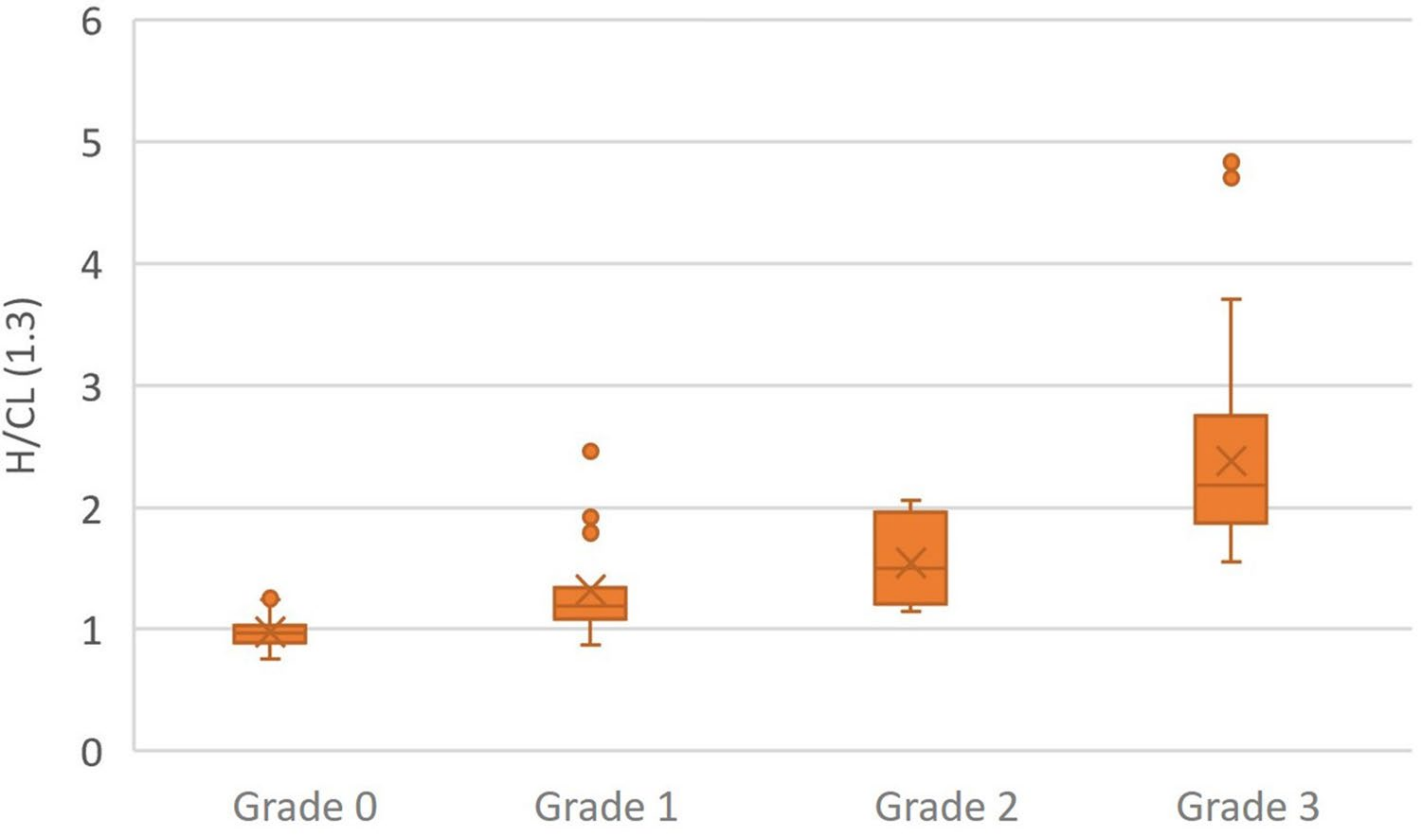
Ranges of values and estimated median uptake for each grade.

It is a quantitative method obtained by calculating the arithmetic mean with their respective standard deviations, extracted from H/CL indices for each Perugini grade. Kruskal Wallis test provided significant differences between individual distributions from each Perugini grade (Table 1) and subsequently identified a range of values for each degree established by the visual score helped improving diagnostic confidence and reduce interobserver variability in a statistically significant way (see Post hoc Analysis in Table 1), with a low standard deviation, ensuring a none statistically significant separation between groups (Table 1, Fig. 3).

**Table 1:** Proposed quantitative score for the diagnosis of CA and statistical analysis.

Kruskal–Wallis				
Chi-squared	129.57	p-value	< 0.001	

Visual score	n	H/CL with a standard deviation	Mean rank	
Grade 0	77	0.98 ± 0.11	41.66	
Grade 1	19	1.32 ± 0.40	83.00	
Grade 2	7	1.54 ± 0.35	100.71	
Grade 3	67	2.40 ± 0.76	135.00	

Visual score	Grade 0	Grade 1	Grade 2	Grade 3
**Post-hoc analysis**				
Grade 0	–	0.006	0.014	< 0.001
Grade 1		–	ns	< 0.001
Grade 2			–	ns
Grade 3				–

The table shows a range of values of H/CL index for each Perugini score with their respective standard deviations, with significant differences between individual distributions (p < 0.05). The Kruskal Wallis test provides significant differences between individual distributions from each Perugini visual score.

**Figure 4:**
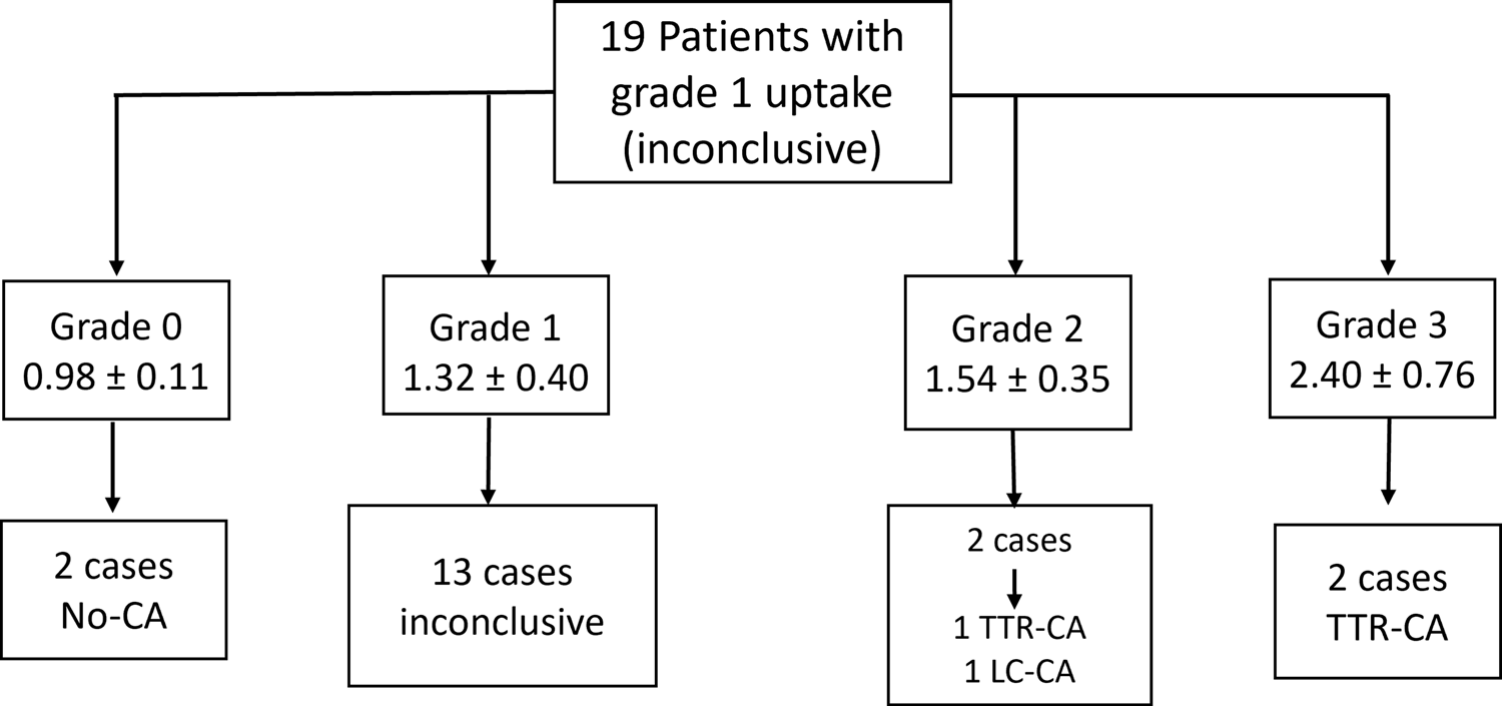
Reclassification of patients with grade 1 uptake with the proposed quantitative method.

When analyzing the results obtained with the proposed quantitative method, a more precise reclassification of patients with grade 1 uptake (inconclusive) was obtained. Thus, of the 19 patients who presented a grade 1 uptake, 2 cases became grade 0 with a final diagnosis of none-CA, 2 became grade 3 with a definitive diagnosis of TTR-CA, and two were regrouped as grade 2, of which one was finally diagnosed with TTR-CA and the other with LC-CA (Fig. 4).

#### H/CL ratio (≥ 1.3 suggestive of TTR-CA and < 1.3 none-CA)

The proposed quantification method supports using a single cut-off value of 1.3 from the H/CL ratio classifying cases into two categories: suggestive or not suggestive of TTR-CA, without inconclusive results. In terms of sensitivity and specificity, the optimization of this cut-off value reclassified four patients previously inconclusive into “suggestive of ATTR-CA”, in which the diagnosis was confirmed in 3 cases and was ruled out in one.

Table 2 shows the sensitivity and specificity values using different H/CL indices thresholds from grade 1 visual score to reclassify doubtful cases, concluding that a 1.3 threshold has the higher sensitivity (94%) without reducing its specificity (p < 0.05).

**Table 2:** Sensitivity and specificity for visual evaluation and quantitative evaluation using three threshold values (1.5, 1.3, and 1.0).

		Sensitivity (%)	Especificificity (%)
Visual score		90	99
Quantitative score	1.5	89	98
	1.3	94	98
	1.0	100	56

#### H/CL ratio (≥ 1.5 suggestive of TTR-CA and < 1 none-CA)

The current diagnostic algorithm classifies 75 patients (44.1%) as “suggestive of TTR-CA”, of which two patients (2.7%) were finally diagnosed of LC-CA, while the remaining were consistent of TTR-CA by international criteria. Also, 45 patients (26.5%) were classified as inconclusive, of which 8 (17.8%) were TTR-CA, 4 (8.9%) LC-CA, and in the remaining 33 patients (73.3%) CA was ruled out. Finally, this method discarded CA in 50 patients (29.4%), of which 3 (6%) were eventually diagnosed with LC-CA.

#### H/CL ratio (1.5 vs 1.3 cut-off value)

**Figure 5:**
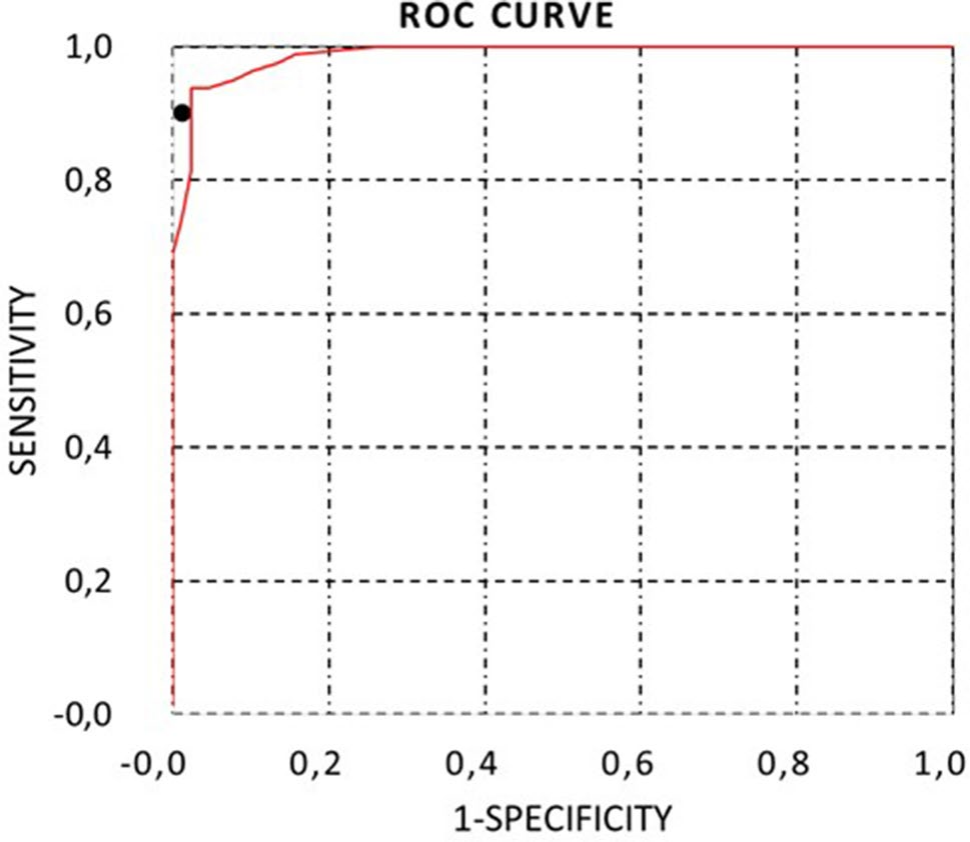
ROC curve of the H/CL index using a cut-off point of 1.3 to classify patients as suggestive of TTR-CA, inconclusive, or not suggestive of TTR-CA. The sensitivity and specificity of the visual evaluation have been represented with a black dot.

From the 45 patients classified as inconclusive with a cut-off point of 1.5, the optimized threshold of 1.3 reclassified 4 more patients as “suggestive of TTR-CA”, rejecting the diagnosis in 41 cases. A 1.5 standard threshold presented an 89% sensitivity and 98% specificity, similar to the visual/semiquantitative evaluation. Still, when a 1.3 threshold is used, the sensitivity increases to 94% without reducing its specificity, with statistically significant differences (Fig. 5).

### Quantitative method (H/CL cut-off point of 1.3) vs. semiquantitative (visual score)

Using the current diagnostic algorithm for CA with a cut-off value of 1.3, the quantitative method classifies a higher percentage of patients as “inconclusive” (N = 41; 24.1%) for the semiquantitative process (N = 19; 11.2%); while the latter rules out the diagnosis of CA in a higher percentage of cases (45.3%) than the H/CL index (29.4%).

Both interpretation methods accurately diagnosed a similar number of TTR-CA patients. The H/CL index correctly diagnosed 76 (93.8%) patients with TTR-CA, and the visual score distributed 73 patients diagnosed with TTR-CA between grades 2 (N = 7; 8.6%) and 3 (N = 66; 81.5%) of myocardial uptake.

## Discussion

While the original validation studies for the Perugini score and the H/CL ratio quoted exceptional sensitivity and specificity for the diagnosis of TTR-CA, there is a growing body of evidence that suggests that the discriminative performance of these methods of analysis of cardiac scintigraphy for TTR-CA is much worse when evaluated in “real-world” populations with a lower pre-test probability of disease than these derived populations at TTR-CA referral centers. Therefore, more patients are classified as equivocal in “real-world” populations than previously reported, and novel methods are desperately needed to refine the non-invasive diagnosis of these patients, given therapeutic advances have made earlier diagnosis more attractive.

The results of the present study show that there are more benefits obtained from the inclusion of ^99^mTc-DPD scintigraphy in the diagnostic algorithm for CA, achieving a correct diagnosis without the use of invasive techniques in 91.3% (N = 74) of all patients with a definitive diagnosis of TTR-CA (N = 81) using the visual score and 93.8% (N = 76) according to the quantitative method (H/CL ≥ 1.3). Likewise, it correctly ruled out CA in 92.5% and 58.8% of the cases according to the semiquantitative and quantitative methods, respectively.

The visual score determined that most patients were grade 3 TTR-CA (81.5%), while most of the LC-CA patients were grade 1 (55.6%) and grade 0 (33.3%). These results correspond with various studies comparing ^99^mTc-DPD scintigraphy with endomyocardial biopsy (gold standard), realizing that TTR-CA expresses great avidity for bone radiotracers, whereas LC-CA expresses minimal or no avidity for these tracers^8^.

Applying the H/CL ratio with a cut-off value of 1.5, as established in the literature, 26.5% were classified as inconclusive, posteriorly reduced to 24.1% with a 1.3 cut-off point. Considering the small number of patients classified as grade 1 in this study (a reflection of the population with CA), it is expected that this slight increase in diagnostic sensitivity provided by the threshold of 1.3, will become more significant with a larger sample. Also, these results should be tested in a prospective and independent model.

It is striking that the quantitative method classified more cases as “inconclusive” than the semiquantitative process (N = 19; 11.1%), similar to the study by Regis et al., who states that the high proportion of inconclusive studies may be related to the lower limit of the threshold (value 1) since a significant amount of activity in the vascular pool of the cardiac cavities can lead to a H/CL index of 1 in most subjects^12^.

The median cardiac uptake using the quantitative score (H/CL ≥ 1.3) showed a directly proportional relationship with the visual method (the higher the degree, the greater the tracer’s cardiac uptake). These results agree with those of the Perugini study and differ from those obtained by Ross et al. in which the median uptake in grade 3 patients was not significantly different from grade 2 subjects or was substantially lower^3^.

It is probably more beneficial to use the C/HC index in the interpretation of planar images acquired at 3 h instead of 1-h post-injection (with a threshold of 1.3), since it decreases the probability of false positives as a consequence of the existence of activity in the vascular pool, cardiomegaly extending to the right of the sternum, or by other anatomical variants, and also the results show a direct relationship with the Perugini grades (higher grade higher index), increasing the degree of concordance between both methods^4,5^.

The results obtained through the interpretation of ^99^mTc-DPD scintigraphy showed that the diagnosis of CA (TTR-CA or LC-CA) is not always clear from the beginning (inconclusive or equivocal studies), despite the advances obtained in the interpretation methods, and continue to suffer the subjectivity of the semiquantitative approach and the lack of a standardized cut-off point for the images acquired 3 h after the administration of ^99^mTc-DPD in the quantitative method.

A 1.3 cut-off is proposed as a more suitable cut-off value when calculating the H/CL ratio in scintigraphic images with ^99^mTc-DPD (3 h post-injection), increasing sensitivity from 89 to 94% without reducing specificity (98%). The relevance of our data relies on the fact that most published studies have a cut-off point of 1.5 in ^99^mTc-PyP scintigraphy after 1-h post-injection^2,4,13^.

Likewise, this additional quantitative method may improve the interpretation obtained in the visual score, reclassifying doubtful cases (grade 1) more accurately and objectively; managing to reduce the percentage of cases classified as grade 1 by 31.5%, regrouping 2, 3, and 1 patient in the categories of non-amyloidosis, TTR-CA, and LC-CA, respectively.

Some of the limitations in this study should be noted, being a retrospective analysis in a unique medical center with a limited number of patients. Thus, it is encouraged to carry studies focused on quantification methods since the proposed cut-off was derived from the same study population in which they were evaluated but might not generalize well to other populations.

## Conclusions

The correct and early classification of patients with suspicion of CA is essential for early treatment, consequently the need to establish enhanced quantification methods. The proposed quantitative method may improve visual scores interpretation, reclassifying doubtful cases (grade 1) more accurately and objectively. Furthermore, optimizing the cut-off value from 1.5 to 1.3 in the quantitative score reclassifies a higher percentage of patients as TTR-CA, with higher sensitivity without reducing its specificity.

## Abbreviations

CA: Cardiac amyloidosis
LC: Light chain
TTR: Transthyretin
LC-CA: Light-chain cardiac amyloidosis
TTR-CA: Transthyretin cardiac amyloidosis
H/CL: Heart–contralateral hemithorax ratio
